# Prognostic value of long noncoding RNA ZFAS1 in various carcinomas: a meta-analysis

**DOI:** 10.18632/oncotarget.21100

**Published:** 2017-09-20

**Authors:** Dan Dong, Zhongyi Mu, Wei Wang, Na Xin, Xiaowen Song, Yue Shao, Chenghai Zhao

**Affiliations:** ^1^ Department of Pathophysiology, College of Basic Medical Science, China Medical University, Shenyang, People's Republic of China; ^2^ Department of Urology, Cancer Hospital of China Medical University, Liaoning Cancer Hospital and Institute, Shenyang, People's Republic of China

**Keywords:** zinc finger antisense 1, long noncoding RNA, prognosis, meta-analysis

## Abstract

A number of studies have revealed that zinc finger antisense 1 (ZFAS1), a long noncoding RNA (lncRNA), is aberrantly regulated in various cancers, and high ZFAS1 expression is associated with poor prognosis and increased risk of lymph node metastasis (LNM). This meta-analysis was conducted to identify the potential value of ZFAS1 as a biomarker for cancer prognosis. We searched electronic database PubMed, Web of Science, and China Wanfang Data (up to June 1, 2017) to collect all relevant studies and explore the association of ZFAS1 expression with overall survival (OS) and LNM. The results showed that cancer patients with high ZFAS1 expression had a worse OS than those with low ZFAS1 expression (HR: 1.94, 95% confidence interval [CI]: 1.41–2.47, *P* < 0.001), and high ZFAS1 expression was significantly associated with LNM (OR: 2.60, 95% CI: 1.54–4.42, *P* < 0.001). Subgroup analysis revealed that high ZFAS1 expression was significantly related to high incidence of LNM in subgroups of sample size more than 88 (OR: 3.16, 95% CI: 2.06–4.86, *P* < 0.001), non-digestive system malignancies (OR: 4.05, 95% CI: 2.49–6.60, *P* < 0.001), and studies reported in 2017 (OR: 4.86, 95% CI: 2.67–8.84, *P* < 0.001) without significant heterogeneity. Further meta-regression by the covariates showed that tumor type, sample size, quality score, cut off value and publication year did not result in the inter-study heterogeneity. In conclusion, the present meta-analysis demonstrates that high ZFAS1 expression may potentially serve as a reliable biomarker for poor clinical outcome in various cancers.

## INTRODUCTION

Cancer is one of the leading causes of morbidity and mortality worldwide today [1]. Although encouraging progress in treatment for cancer has been achieved, the 5-year survival rate remains low and the majority of patients die due to relapse and metastases [2]. The ideal prognostic marker helps predict the tumor prognosis, which is of great significance for designing reasonable plans for disease surveillance and treatment. Recently, researchers focused on tumor biomarkers and have identified numerous potential biomarkers for tumor prognosis and treatment [3–4]. However, it is urgently needed for the sensitive and specific biomarkers for prognosis of patients with cancers.

Long noncoding RNAs (lncRNAs) are nonprotein-coding transcripts with length of 200 nucleotides, which have been dismissed as transcriptional “noise” in the past decade [5, 6]. Nowadays, accumulating evidence shows that lncRNAs play tremendous roles in cell proliferation, differentiation, apoptosis and transformation [7–9]. Clinicopathologic studies have suggested that lncRNAs are related to prognosis and metastasis of various tumors, therefore, they may be sensitive and specific biomarkers for the prediction of cancer progression and prognosis [10–13].

Zinc finger antisense 1 (ZFAS1) is a newly identified lncRNA, which has attracted widespread attention recently. Aberrant high expression of ZFAS1 was reported in a series of human cancers, including hepatocellular carcinoma [14], gastric cancer [15–17], melanoma [18], lung cancer [19], glioma [20] and colorectal cancer [21–23]. It was observed that high ZFAS1 expression was associated with metastasis and prognosis, thus ZFAS1 may be a potential biomarker for prognosis. This current meta-analysis was performed to explore the correlation of ZFAS1 expression with clinical outcome of cancer patients, and further determine whether ZFAS1 could serve as an effective biomarker for metastasis and prognosis.

## MATERIALS AND METHODS

### Search strategy

We searched for potentially eligible literatures published up to June 2017 through PubMed, Embase, Web of Science, Chinese Wan Fang and CNKI database for the meta-analysis. The search strategy used both MeSH terms and free-text words to increase sensitivity. The keywords for the search were as follows: “ZFAS1 and cancer”, “long non-coding RNA ZFAS1”, “lncRNA ZFAS1”, “ZFAS1”. Articles were limited to English-language and Chinese-language publications. Meanwhile, reference lists of relevant articles were also reviewed to identify potentially eligible papers.

### Inclusion and exclusion criteria

In this meta-analysis, eligible studies had to meet the following standards: 1) studies with a cohort design, 2) studies investigating the correlation between ZFAS1 expression and cancer patients, 3) studies in which ZFAS1 expression in primary tumor tissues was measured by real-time quantitative reverse transcription PCR (qRT-PCR), 4) studies with sufficient original data for calculating odd ratios (ORs), hazard ratios (HRs) and their 95% confidence interval (95% CI). If an article only provided survival curves without offering HR and 95% CI directly, appropriate data were extracted from the survival curves using Engauge Digitizer 4.1 software, and logHR and selogHR were calculated according to Tierney *et al*. [24]. The following criteria were used to exclude studies: 1) duplicate publications; 2) studies of case reports, letters, and reviews; 3) studies without usable data.

### Date extraction

Two investigators extracted the data independently through a same standard. Any disagreement was consulted with a third investigator. The following details were extracted: first author, publication year, country of origin, cancer type, detection method of ZFAS1, total number of patients, number of high ZFAS1 expression group and low expression group, number of patients with LNM, the HR and the corresponding 95% confidence interval (CI) for overall survival (OS).

### Statistical analysis

ORs and 95% CI were used to evaluate the relationship between ZFAS1 expression and LNM, and HRs and 95% CI were used to assess the effect of ZFAS1 expression on the survival. In order to evaluate the heterogeneity of the included studies, Cochrane *Q*-test and I^2^ statistics were performed by using Stata 12.0 Software (Stata, College Station, TX, USA). If there was a significantly statistical heterogeneity (I^2^ ≥ 50% or *P* ≤ 0.05) among the studies, we used the random-effects model to analyze the results, and performed subgroup and sensitivity analysis to dissect the heterogeneity. If the heterogeneity was absent, the fixed-effects model was applied to this meta-analysis. In addition, the Stata 12.0 Software was used to evaluate the sensitivity of the studies. Publication bias was evaluated by Begg's test and Egger's test, *P* < 0.05 was considered statistically significant. Forest plots were used to dispaly the meta-analysis results, and Begg's funnel plots were used to show publication bias.

The method reported by Wacholder et al. [25] was used to analyze the false positive report probability (FPRP) and statistical power of each significant correlation. A prior probability of 0.1 was set to detect an OR of 0.67/1.50 (protective/risk effects). When the FPR*P* value was lower than 0.2, the correlation was noteworthy. Statistical power and FPR *P* value were calculated using the Excel spreadsheet provided by Wacholder et al. [25].

## RESULTS

### Characteristics of eligible studies

**Figure 1:**
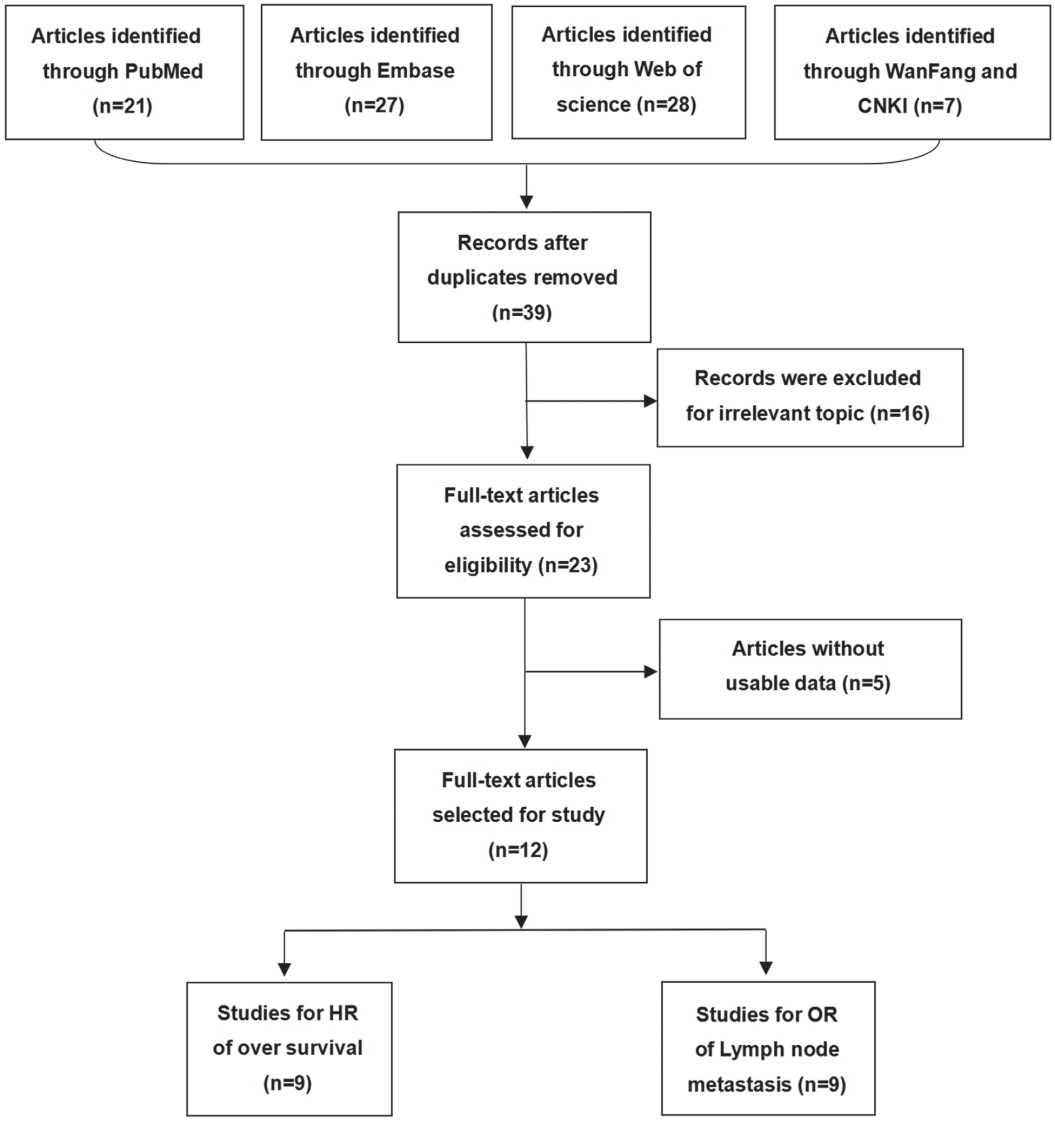
Flowchart presenting the steps of literature search and selection

A flow diagram of literature search process was presented in Figure 1. After searching PubMed, Embase, Web of Science, Chinese Wan Fang and CNKI database, we selected twelve studies ranging 2015 to 2017 based on the inclusion criteria, and these studies were all from People's Republic of China. Seven different types of cancer were evaluated in the meta-analysis, with one case of non-small cell lung cancer (NSCLC), three cases of gastric cancer (GC), three cases of colorectal cancer (CRC), one case of hepatocellular carcinoma (HCC), two case of glioma, one case of melanoma and one case of ovarian cancer (OC).

Table 1 summarized the main characteristics of the included twelve studies enrolling 1075 participants, with the mean patient sample size of 80.5 (range 54–173). In these studies, ZFAS1 expression in tumor tissues was determined by qRT-PCR. All studies comprised a high ZFAS1 expression group and a low ZFAS1 expression group. Nine studies investigated the association between ZFAS1 expression and OS, and nine studies assessed the association between ZFAS1 expression and LNM. HRs and 95% CIs were directly extracted from five studies, and calculated by survival curves in five studies. ORs and 95% CIs were directly extracted from nine studies.

**Table 1:** Characteristics of ZFAS1 studies included in the meta-analysis

First auhor	Year	Country	Cancer Type	Sample size	ZFAS1 expression				Detection method	Cut-off value	Outcome	HR estimate	NOS score	IF value
					High with LNM	High without LNM	Low with LNM	Low without LNM						
Wang et al.	2016	China	CRC	159	50	29	38	42	qRT-PCR	Median	OS	Reported	7	1.543
Tian et al.	2016	China	NSCLC	173	58	27	34	54	qRT-PCR	NA	OS	Reported	6	1.778
Nie et al.	2016	China	GC	54	12	15	16	11	qRT-PCR	Median	OS	Survival curve	8	5.168
Pan et al.	2017	China	GC	94	48	10	19	17	qRT-PCR	NA	-	-	6	3.502
Fang et al.	2016	China	CC	73	15	21	6	31	qRT-PCR	NA	OS	Survival curve	7	3.452
Lv et al.	2017	China	glioma	69	-	-	-	-	qRT-PCR	Median	OS	Reported	6	3.650
Li et al.	2015	China	HCC	88	-	-	-	-	qRT-PCR	Median	OS	Survival curve	7	9.122
Wu et al.	2016	China	CRC	67	12	22	15	18	qRT-PCR	Median	-	-	6	NA
Wei et al.	2017	China	melanoma	88	38	8	22	20	qRT-PCR	Median	OS	Reported	8	1.706
Zhang et al.	2016	China	GC	104	25	27	8	44	qRT-PCR	Median	OS	Reported	8	1.706
Xia et al.	2017	China	OC	60	16	14	4	26	qRT-PCR	NA	OS	Survival curve	7	5.168
Gao et al.	2017	China	glioma	46	-	-	-	-	qRT-PCR	NA	OS	Survival curve	6	1.971

### Relationship between ZFAS1 and OS

**Figure 2:**
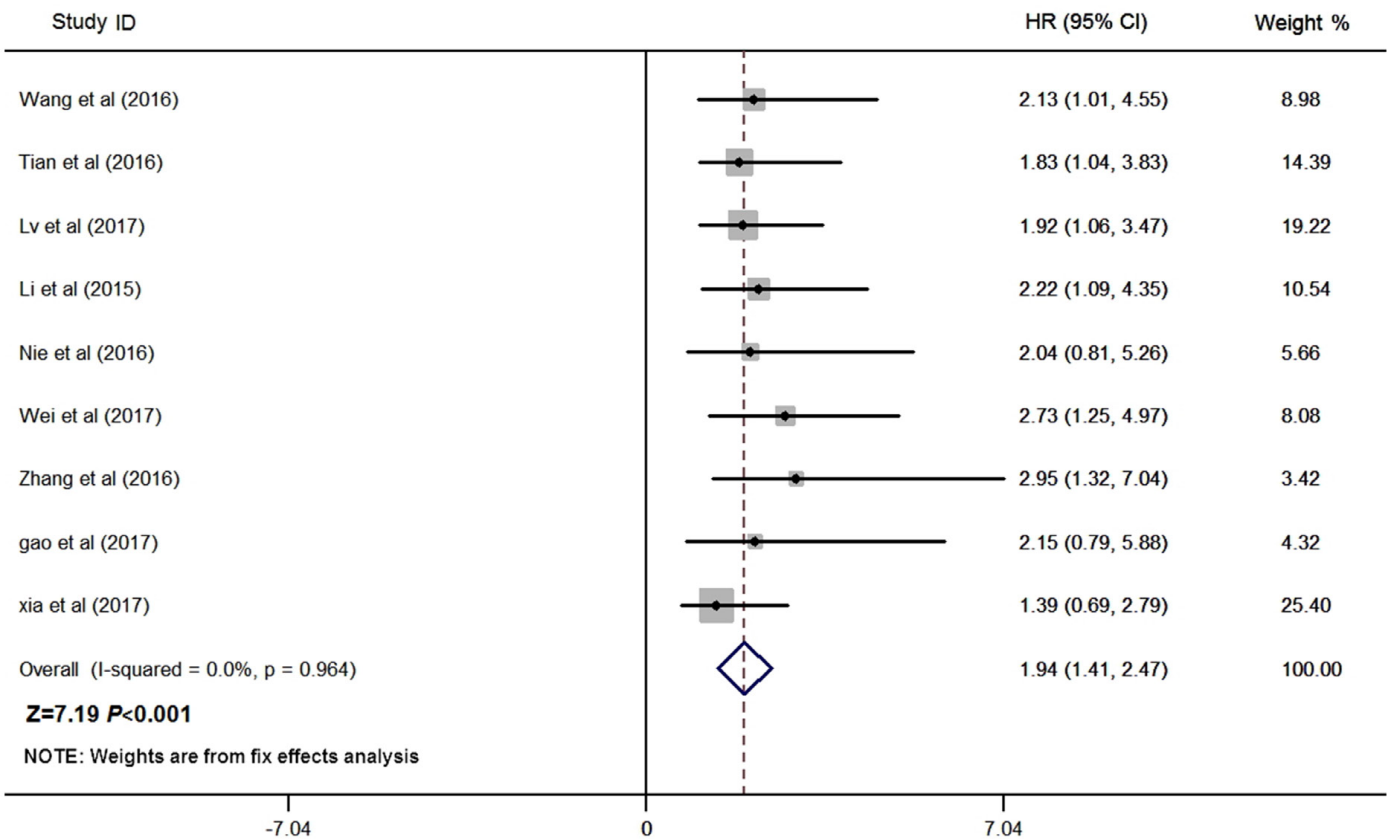
Forest plot for the association between ZFAS1 expression with OS

Nine studies consisting of 841 patients reported the OS according to levels of ZFAS1 expression, and the median sample size was 88 (range 54–173) in this meta-analysis. Given that there was no heterogeneity across these studies (I^2^ = 0.0%, *P* = 0.964; Figure 2), the fixed-effects model was applied to estimate the pooled HRs and the respective 95% CIs. As shown in Figure 2, our results revealed that high ZFAS1 expression predicted poor OS in various cancers (pooled HR: 1.94, 95% CI: 1.41–2.47, *P* < 0.001; fixed-effect).

### Relationship between ZFAS1 and LNM

**Figure 3:**
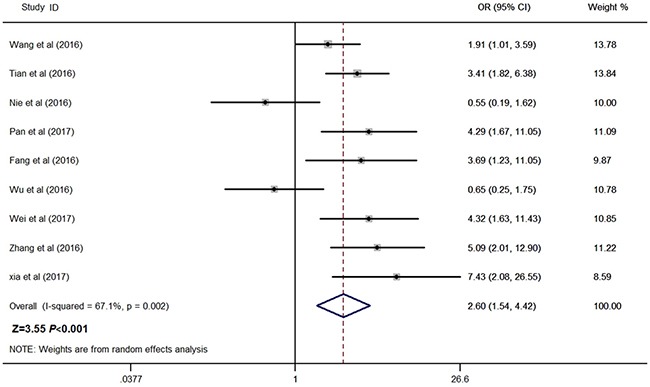
Forest plot for the association between ZFAS1 expression with LNM

**Figure 4:**
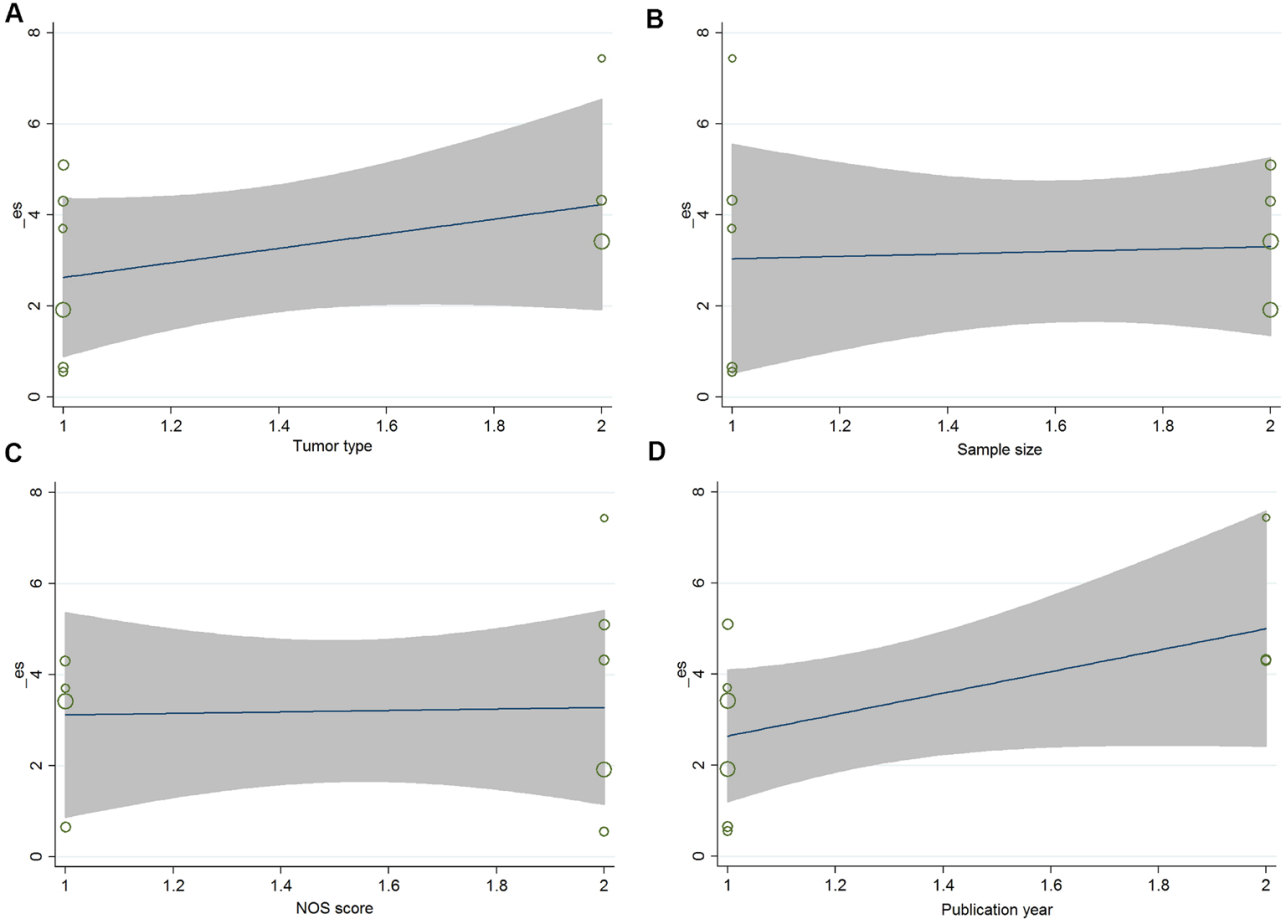
(**A**) Based on tumor type ; (**B**) Based on sample size; (**C**) Based on NOS score; (**D**) Based on publication year.

Nine studies reported the number of patients with LNM in a total of 872 individuals. The median sample size was 88 (range 54–173). As shown in Figure 3, the random-effects model was adopted for the significant heterogeneity (I^2^ = 67.1%, *P* = 0.002). Our results revealed that high ZFAS1 expression was significantly associated with LNM (pooled OR: 2.60, 95% CI: 1.54–4.42, *P* < 0.001, random-effects). Because of the significant heterogeneity between studies, subgroups were analyzed based on the tumor type, sample size, quality score and publication year (Table 2). Our data revealed that high ZFAS1 expression was related to high incidence of LNM in subgroups of sample size more than 88 (OR: 3.16, 95% CI: 2.06–4.86, *P* < 0.001), non-digestive system malignancies (OR: 4.05, 95% CI: 2.49–6.60, *P* < 0.001), studies reported in 2017 (OR: 4.86, 95% CI: 2.67–8.84, *P* < 0.001) without significant heterogeneity. In addition, we further performed meta-regression by the covariates including tumor type, sample size, quality score and publication year. As shown in Table 2 and Figure 4, those factors did not result in the inter-study eterogeneity.

**Table 2:** Results of subgroup analysis of increased ZFAS1 expression and LNM in various carcinomas

Stratified analysis	No. of studies	No. of patients	Heterogeneity		Pooled OR (95% CI)	*P*-value	Meta-regression *P* value
			I_2_ (%)	*P* value			
Tumor type							0.144
Digestive system	6	551	72.3	0.003	1.98 (0.97–4.04)	0.062	
Non-digestive system	3	321	0	0.555	4.05 (2.49–6.60)	< 0.001	
Sample size							0.825
Number ≤ 88	4	342	77.9	0.001	2.07 (0.74–5.74)	0.164	
Number > 88	5	530	23.2	0.272	3.16 (2.06–4.86)	< 0.001	
NOS score							0.610
≤ 6	4	407	59.7	0.059	1.51 (1.06–2.16)	0.023	
> 6	5	465	75.3	0.003	1.62 (1.06–2.48)	0.027	
Publication year							0.069
2016	6	630	72.6	0.003	1.94 (1.00–3.78)	0.050	
2017	3	242	0.0	0.002	4.86 (2.67–8.84)	< 0.001	

**Figure 5:**
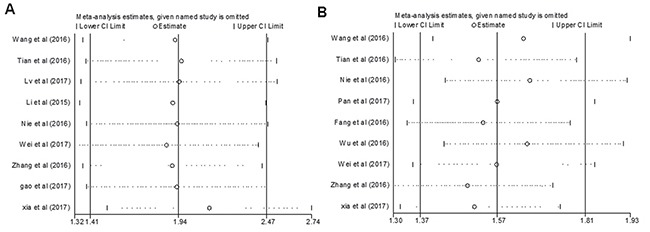
Sensitivity analyses of the studies (**A**) Overall survival; (**B**) Lymph node metastasis.

### FPRP test

The significant associations were investigated by using the FPRP test. For a prior probability of 0.1, high ZFAS1 expression was associated with LNM in subgroup of nondigestive system malignancies (FPRP < 0.001), sample size more than 88 (FPRP < 0.001) and studies reported in 2017 (FPRP < 0.001). FPR*P* value and statistical power were shown in Table 3.

**Table 3:** False-positive report probability values for associations between increased ZFAS1 expression and LNM in various carcinomas

Stratified analysis	Pooled OR (95% CI)	*P*-value	Statistical Power	Prior probability				
				0.25	0.1	0.01	0.001	0.0001
Tumor type								
Digestive system	1.98 (0.97–4.04)	0.062	0.564	0.243	0.491	0.914	0.991	0.999
Non-digestive system	4.05 (2.49–6.60)	< 0.001	0.520	< 0.001	< 0.001	< 0.001	< 0.001	< 0.001
Sample size								
Number ≤ 88	2.07 (0.74–5.74)	0.164	0.511	0.471	0.727	0.967	0.997	1.000
Number > 88	3.16 (2.06–4.86)	< 0.001	0.523	< 0.001	< 0.001	< 0.001	< 0.001	0.003
NOS score								
≤ 6	1.51 (1.06–2.16)	0.023	0.485	0.129	0.308	0.831	0.980	0.998
> 6	1.62 (1.06–2.48)	0.027	0.362	0.180	0.396	0.878	0.986	0.999
Publication year								
2016	1.94 (1.00–3.78)	0.050	0.536	0.224	0.464	0.905	0.990	0.999
2017	4.86 (2.67–8.84)	< 0.001	0.537	< 0.001	< 0.001	< 0.001	< 0.001	0.004

### Sensitivity analysis and publication bias

**Figure 6:**
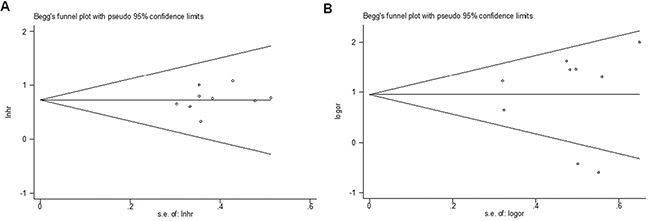
Begg's test for publication bias (**A**) Overall survival; (**B**) Lymph node metastasis. Abbreviations: SE, standard error.

Sensitivity analysis was performed by using Stata12.0 software to assess whether the individual study affected the overall results. The results showed that individual study had little influence on our final results (Figure 5), thus our results were relatively stable and credible. We used both Begg's test and Egger's test to evaluate publication bias. Begg's funnel plot with pseudo 95% CIs was provided. Our data didn't revealed publication bias across the studies, including meta-analysis of the association of ZFAS1 expression with OS (Begg's test:Pr>|Z| = 0.602; Egger's test: P>|t| = 0.459) and LNM (Begg's test: Pr >|Z| = 0.917; Egger's test:P >|t| = 0.949) (Figure 6).

## DISCUSSION

Recent studies indicated that numberous lncRNAs such as nuclear paraspeckle assembly transcript 1 (NEAT1), cyclin D2 antisense RNA 1 (CCND2-AS1), taurine-upregulated gene 1 (TUG1) and HOX transcript antisense RNA (HOTAIR) were overexpressed in tumors, and involved in tumor occurrence and progression [26–29]. As potential new molecular biomarkers, differential levels of lncRNAs or cancer-specific lncRNA profiles may be used for the prediction of cancer prognosis.

ZFAS1, located at chromosomal band 20q13.13, was first reported dysregulated in breast cancer, suggesting a role of ZFAS1 in this type of cancer [30]. Subsequently, ZFAS1 was confirmed involved in a series of human tumors. For instance, Li *et al*. showed that ZFAS1 was amplified in HCC, and promoted HCC cell invasion and migration by positively regulating ZEB1, MMP14 and MMP16 [14]. Nie *et al*. found that ZFAS1 might act as a oncogene in GC by repressing KLF2 and NKD2 expression [15]. Moreover, Wang *et al*. showed that ZFAS1 was upregulated in CRC, which prompted metastasis of CRC [22]. Recently, studies indicated consistent results about the relationship between high ZFAS1 expression and poorer prognosis in CRC, GC, HCC, glioma, melanoma and NSCLC [14, 15, 17, 18, 19, 20, 22]. However, there is controversy about the relationship between ZFAS1 expression and LNM in various cancers. Some studies indicated that high ZFAS1 expression was associated with LNM [16, 17, 18, 19, 21, 22], While others showed no significant associated between elevated ZFAS1 levels and LNM [15, 23]. These findings suggest that ZFAS1 may be a promising indicator of prognosis in human cancers, but further confirmation by extensive analysis is required.

In our meta-analysis, we assessed the association of ZFAS1 expression with metastasis and prognostic outcome. From the available studies, we found that high ZFAS1 expression was associated with poor OS in different types of cancers without significant heterogeneity, suggesting that ZFAS1 may serve as a reliable molecular marker for poor prognosis in various cancers. In addition, we found there was a significant association between ZFAS1 expression and LNM, but with heterogeneity. Therefore, we performed subgroup analysis to precisely assess the association of ZFAS1 with LNM. Subgroup analyses showed a remarkable decrease in the heterogeneity of LNM in subgroups of “sample size more than 88”, “non-digestive system malignancies’ and “studies reported in 2017”, suggesting that “tumor type” , “sample size” and “publication year ” may be as sources of heterogeneity. Moreover, data from subgroup analysis showed that high ZFAS1 expression was related to high incidence of LNM in the subgroup of sample size > 88, non-digestive system malignancies and studies reported in 2017, which further strengthened the pooled result. To make our results more reliable, we performed FPRP test, and found that the correlation of high ZFAS1 expression with LNM in subgroup of nondigestive system malignancies, sample size > 88 and studies reported in 2017 all passed the FPRP test, indicating these associations were reliable.

Taken together, our meta-analysis results indicate that ZFAS1 may act as a novel biomarker in predicting the clinical outcome of cancer patients. Further studies exploring the relationship between LNM and ZFAS1 expression are required to verify its clinical prognostic value in human cancers.

There are some limitations in our analysis. First, the number of patients and types of cancers included are relatively small. Second, studies included in the meta-analysis all come from People's Republic of China, for this reason, our results may just represent the cases of Chinese cancer patients. Third, positive results can be published more easily than negative ones, which may lead to larvaceous publication bias. Fourth, this meta-analysis is a retrospective analysis, which may limit the conclusion due to selection bias. Fifth, the quality of studies included in the meta-analysis was uneven and thus limit the precision and generalizability of the pooled estimates. Finally, these studies lack a unified criterion for high ZFAS1 expression. Therefore, larger-scale, multicenter, and higher-quality studies are required to confirm our findings in the future.
